# Umbilical Cord Blood IL-6 as Predictor of Early-Onset Neonatal Sepsis in Women with Preterm Prelabour Rupture of Membranes

**DOI:** 10.1371/journal.pone.0069341

**Published:** 2013-07-24

**Authors:** Teresa Cobo, Marian Kacerovsky, Ctirad Andrys, Marcela Drahosova, Ivana Musilova, Helena Hornychova, Bo Jacobsson

**Affiliations:** 1 Maternal Fetal Medicine Department, Hospital Clinic, Institut d' Investigacions Biomèdiques August Pi i Sunyer (IDIBAPS), Universitat de Barcelona, Barcelona, Spain; 2 Biomedical Research Center, University Hospital Hradec Kralove, Hradec Kralove, Czech Republic; 3 Department of Obstetrics and Gynaecology, University Hospital Hradec Kralove, Hradec Kralove, Czech Republic; 4 Department of Immunology, University Hospital Hradec Kralove, Hradec Kralove, Czech Republic; 5 Department of Obstetrics and Gynecology, Hospital Pardubice, Pardubice, Czech Republic; 6 Fingerland's Department of Pathology, University Hospital Hradec Kralove, Hradec Kralove, Czech Republic; 7 Department of Obstetrics and Gynecology, Sahlgrenska University Hospital, Gothenburg, Sweden; 8 Institute of Public Health, Oslo, Norway; 9 Centro de Investigación Biomédica en Red de Enfermedades Raras (CIBERER), Barcelona, Spain; John Hunter Hospital, Australia

## Abstract

**Objective:**

To evaluate umbilical cord interleukin (IL)-6 and funisitis as independent predictors of early-onset neonatal sepsis (EONS) in preterm prelabor rupture of membranes (PPROM).

**Design:**

Prospective cohort study.

**Setting:**

Evaluation of umbilical cord IL-6 and funisitis as predictors of early-onset neonatal sepsis in PPROM.

**Population:**

176 women with PPROM between 23+0−36+6 weeks of gestation.

**Methods:**

Umbilical cord IL-6 was assayed by ELISA. Funisitis was defined according to the Salafia classification. Data was adjusted by gestational age at delivery and prenatal administration of corticosteroids and antibiotics.

**Main Outcome Measures:**

Binary logistic regression was performed to assess the independence of umbilical cord IL-6 and funisitis to predict EONS in women complicated with PPROM.

**Results:**

The rate of EONS was 7%. Funisitis was present in 18% of women. Umbilical cord IL-6 was significantly higher in women complicated with EONS than without [median (range) 389.5 pg/mL (13.9–734.8) vs 5.2 (0.1–801–4), *p*<0.001]. Umbilical cord IL-6 was the only independent predictor of early-onset neonatal sepsis (odds ratio 13.6, *p* = 0.004).

**Conclusion:**

Umbilical cord IL-6 was the only predictor of early-onset neonatal sepsis in PPROM. Contrary to what is reported, funisitis was not.

## Introduction

Early-onset neonatal sepsis (EONS) is a serious complication with a mortality rate ranging from 1.5% in term to almost 40% in very-low-birth weight infants [1]. Neonatal symptoms and laboratory markers of neonatal sepsis may be indistinguishable from various non-infectious conditions. No currently available test is able to provide perfect diagnostic accuracy, and false-negative as well as false-positive results may occur. Thus, empirical antibiotic therapy is current practice in all neonates with suspicion of EONS. However, empirical treatment also increases the exposure to adverse drug effects, nosocomial complications and a high risk to develop resistant strains. An early prediction of EONS could improve neonatal management of newborns complicated with EONS.

Fetal inflammatory response syndrome (FIRS) is the fetal response to intra-uterine infection where there is an activation of inflammatory mediators with defence functions. FIRS has been defined by high levels of pro-inflammatory cytokines in fetal blood [2]–[7]. One of these pro-inflammatory mediators, interleukin (IL)-6, has been considered the best marker of neonatal morbidity [7]–[9]. Thus, IL-6 is postulated to be an inflammatory marker of EONS, particularly in preterm neonates [10].

From a histopathology point of view, FIRS is also defined as funisitis. Funisitis is a polymorphonuclear leukocyte infiltration along the umbilical cord in response to infection. It is considered the last stage of intra-uterine infection responsible for a worse neonatal outcome [11]–[13], including the risk of EONS.

Despite umbilical cord blood IL-6 and funisitis have been classically reported as independent predictors of neonatal morbidity, gestational age at delivery is considered determinant of global neonatal morbidity prognosis. Thus, advances in perinatology, such as prenatal steroids and antibiotic exposure or surfactant administration in neonatal management, have contributed to a considerable improvement in neonatal outcomes. This is why the aim of the present study is to determine whether umbilical cord blood IL-6 and funisitis remain independent markers of EONS regardless current pre- and postnatal management of women with preterm prelabor rupture of membranes (PPROM). We hypothesize that the postnatal information of the umbilical cord blood IL-6 value and the occurrence of funisitis could be useful for clinical neonatal management to predict the high-risk group of EONS.

## Methods

A prospective cohort study was performed in pregnant women between 23+0 and 36+6 weeks of gestation with a PPROM diagnosis who were admitted to the Department of Obstetrics and Gynecology, University Hospital Hradec Kralove, Czech Republic, between July 2008 and October 2010. Gestational age was established according to the first-trimester ultrasound scan. Multiple pregnancies, structural/chromosomal anomalies and patients with clinical signs of chorioamnionitis or vaginal bleeding at admission were not considered eligible for this study.

PPROM was defined as leakage of amniotic fluid that precedes the onset of uterine contractions and cervical changes. PPROM was diagnosed by a sterile speculum examination to identify pooling of amniotic fluid in the vagina in association with a positive test for the presence of insulin-like growth factor–binding protein (ACTIM PROM test; MedixBiochemica, Kauniainen, Finland) in the vaginal fluid.

A complete course of antenatal steroids, which included intramuscular injection of betamethasone 12 mg with two doses given 24 h apart, was administered when PPROM occurred from 24+0 to 33+6 weeks. Tocolysis was considered for 48 h in the absence of clinical chorioamnionitis, abruptio placentae and fetal compromise. Prophylactic parenteral broad-spectrum antibiotics with azithromycin were given at admission during 7 days. No treatment, except antibiotics, was initiated to delay delivery after 34 weeks. Management of PPROM women in the Czech Republic is active except for PPROM pregnancies at <28 weeks of gestation, which are handled with expectant care. The timeline for inducing labour or elective caesarean section depends on gestational age: within 24 hours for those with gestational age above 34+0 weeks, within 48 hours for those between 32+0 and 33+6 weeks of gestation, and within 72 hours after rupture of the membranes for those between 28+0 and 31+6 weeks [14].

Fetal and maternal statuses were closely monitored until delivery. Maternal serum C-reactive protein (CRP) level and white blood cell (WBC) count were assayed upon admission and every subsequent day until delivery.

After delivery, the placenta, the fetal membranes and the umbilical cord were fixed in 10% neutral buffered formalin. Tissue samples obtained from the placenta (at least 2 samples), umbilical cord (usually 1 sample) and placental membranes (at least 2 samples) were routinely processed and embedded in paraffin. Sections of tissue block were stained with hematoxylin and eosin for a standard histological examination. Histopathological examinations were performed by a single pathologist who was blinded to the clinical status of the patients. The degree of polymorphonuclear leukocyte infiltration was evaluated separately in the free membranes (amnion and chorion-decidua), the chorionic plate and the umbilical cord according to criteria proposed by Salafia et al [15]. The diagnosis of funisitis was determined based on grades 1–4 in the umbilical cord.

Blood samples were obtained from clamped umbilical cords after delivery of the neonates and prior to the delivery of the placenta. Samples were collected using a vacutainer blood collecting system and then centrifuged. Supernatants were stored in polypropylene tubes at −70°C until testing. Enzyme-linked immunosorbent assays (ELISA) for human IL-6 were performed for umbilical cord blood samples (R&D Systems Inc., USA). The sensitivity of test was less than 0.70 pg/mL, and inter-assay and intraassay coefficients were less than 10%.

For all new-borns, data records regarding gestational age at delivery, morbidity and mortality were reviewed. EONS was defined as the presence of confirmed or suspected sepsis at ≤72 hours after birth. Confirmed sepsis represented the presence of a positive blood culture. A diagnosis of suspicion of EONS was based in the presence of clinical symptoms (e.g. body temperature inestability, hypotension, poor perfusion with pallor, tachycardia or bradycardia, apnoea, respiratory distress, grunting, cyanosis, irritability, lethargy, seizures, refusal to feed, abdominal distension, petechial, purpura and bleeding) corroborated with two or more hematological laboratory results in the absence of a positive blood culture [16]–[17]: (1) absolute neutrophil count of <7500 or >14 500 cells/mm^3^, (2) absolute band count >1500 cells/mm^3^, (3) immature/total (I: T) neutrophil ratio >0.16, (4) platelet count <150 000 cells/mm^3^ [18].

Written informed consent was obtained from all subjects. The women in this study have previously been part of another more extensive publication studying histologic chorioamnionitis and funisitis in relation to a broader neonatal outcome. The present publication deals with a subcohort were both information about funisitis and IL-6 in the umbilical cord was present [19].

### Ethics Statement

The Institutional Review Board approved the collection and use of these samples and information for research purposes (Ethics committee of University Hospital Hradec Kralove, Sokolska 581, 500 05, Hradec Kralove. March 19, 2008; No. 200804 SO1P). Written informed consent was obtained from all subjects.

### Statistical analysis

Statistical analyses were performed using SPSS 19.0 for Windows XP OS (SPSS Inc., Chicago, IL, USA). Demographic and clinical characteristics were compared using the nonparametric Mann-Whitney U test, and the results are presented as medians (range). Categorical variables were compared using the linear-by-linear association Chi-squared test and are presented as percentages (%). Receiver operator curve (ROC) analysis was employed to display the relationship between sensitivity and false positive (FP) rate (1-specificity) and to select the best cut-off value for umbilical cord IL-6 to predict EONS. Binary logistic regression was performed to assess the independence of umbilical cord blood IL-6 and funisitis to predict early-onset neonatal sepsis. Data was adjusted by gestational age at delivery, administration of prenatal corticosteroids and antibiotics. Differences were considered statistically significant at a confidence level of *p* <0.05 with two-sided alternative hypotheses.

## Results

Between July 2008 and October 2010, 176 women with a diagnosis of PPROM between 23+0 and 36+6 weeks of gestation were admitted to the department and met inclusion criteria. Median gestational ages at sampling and at delivery for the entire study population were 32+6 weeks (range: 24–36+5) and 33+2 weeks (range: 24–36+6), respectively. In 6 cases, the placenta could not be retrieved for histological examination. The overall rate of funisitis was 18% (30/170). There were no differences on corticosteroids and/or antibiotic administration among groups.

Microbial invasion of the amniotic cavity (MIAC) was present in 36% of women (63/176). The rate of EONS in our study was 7% (12/176). Four newborn complicated with EONS presented a positive hemoculture. Microorganisms isolated were Streptococcus α-hemolyticus (n = 1), Escherichia coli (n = 1), Hemophilus influenzae (n = 1) and Streptococcus pneumoniae (n = 1). Latency from delivery to suspicion of EONS was median (range) 1 day (1–2) [median (range)]. Seven of cases had the funisitis with either grade 3 (neutrophils in the perivascular Wharton jelly) or grade 4 (panvasculitis and funisitis deep into the Wharton jelly). Four of cases had histological chorioamnionitis with grade 4 in chorionic plate (numerous neutrophils in chorionic plate and chorionic vasculitis) and neutrophils infiltrations of amniotic epithelium. Only one case of EONS (cultivation proven – Streptococcus pneumoniae) was without histological chorioamnionitis.

Maternal and neonatal characteristics according to the presence or absence of EONS are summarised in Table 1 and 2, respectively. Gestational age at PPROM and delivery were significantly lower in women with EONS. The levels of umbilical cord IL-6 and the occurrence of funisitis were significantly higher in PPROM women with EONS than without. The occurrence of EONS was significantly higher in women with microbial invasion of the amniotic cavity (MIAC) than in women without. No other significant differences were observed for maternal or neonatal outcomes according to the presence or absence of EONS.

**Table 1:** Maternal outcomes according the presence or absence of early-onset neonatal sepsis.

	Presence EONS n 12	Absence EONS n 164	*p*
Maternal age	28 (20–37)	31 (18–44)	0.131
Body mass index	20.1 (17–31.2)	22.1 (16.3–40.6)	0.113
Smoking	4 (33.3)	21 (12.8)	0.228
Nuliparity	2 (16.7)	71 (43.3)	0.013
Corticosteroids	8 (66.7)	52 (31.7)	0.363
Antibiotics	11 (91.7)	100 (61)	0.538
GA at PPROM (weeks)	30.5 (25–35)	33 (24–36)	0.013
CRP (mg/L)	3.6 (0–22)	6.6 (0–82)	0.265
Maternal WBC count at admission (x10^9^/L)	11.95 (7–19)	12 (7–26.8)	0.643
MIAC	8 (67)	42 (26)	0.041
GA at delivery (weeks)	30.5 (25–35)	34 (24–36)	0.006
Latency PPROM-Delivery (h)	52 (8–244)	32 (5–242)	0.309
Funisitis	7 (58.3)	14 (8.5)	0.000
UC IL-6 (pg/mL)	389.5 (13.9–734.8)	4.1 (0.1–801.4)	0.000

GA: Gestational age; PPROM: Preterm pre-labour rupture of membranes, CRP: C-reactive protein, WBC: White Blood count, UC: Umbilical cord. MIAC: Microbial invasion of the amniotic cavity.

Continuous variables were compared using a nonparametric Mann-Whitney U test presented as median (range). Categorical variables were compared using Chi-square test and presented as number (%).

**Table 2:** Neonatal outcomes according the presence or absence of early-onset neonatal sepsis.

	Presence EONS n 12	Absence EONS n 164	*p*
Birthweight (g)	1775 (710–2870)	1940 (490–3870)	0.167
Positive newborn hemoculture	4 (33)	0	0.000
Newborn WBC count (×10^9^/L)	10.5 (2–29)	13 (5–41)	0.090
Respiratory distress syndrome	5 (42)	31 (19)	0.298
Intraventricular haemorrage	3 (25)	32 (19)	1.000
Necroziting enterocolitis	0	2 (1)	1.000
Pulmonary broncodisplasia	1 (8)	7 (4)	0.524
Retinopathy	1 (8)	8 (5)	0.567
Fetal death	1 (8)	1 (0.6)	0.166

WBC: White Blood count. Continuous variables were compared using a nonparametric Mann-Whitney U test presented as median (range). Categorical variables were compared using Chi-square test and presented as number (%).

**Figure 1:**
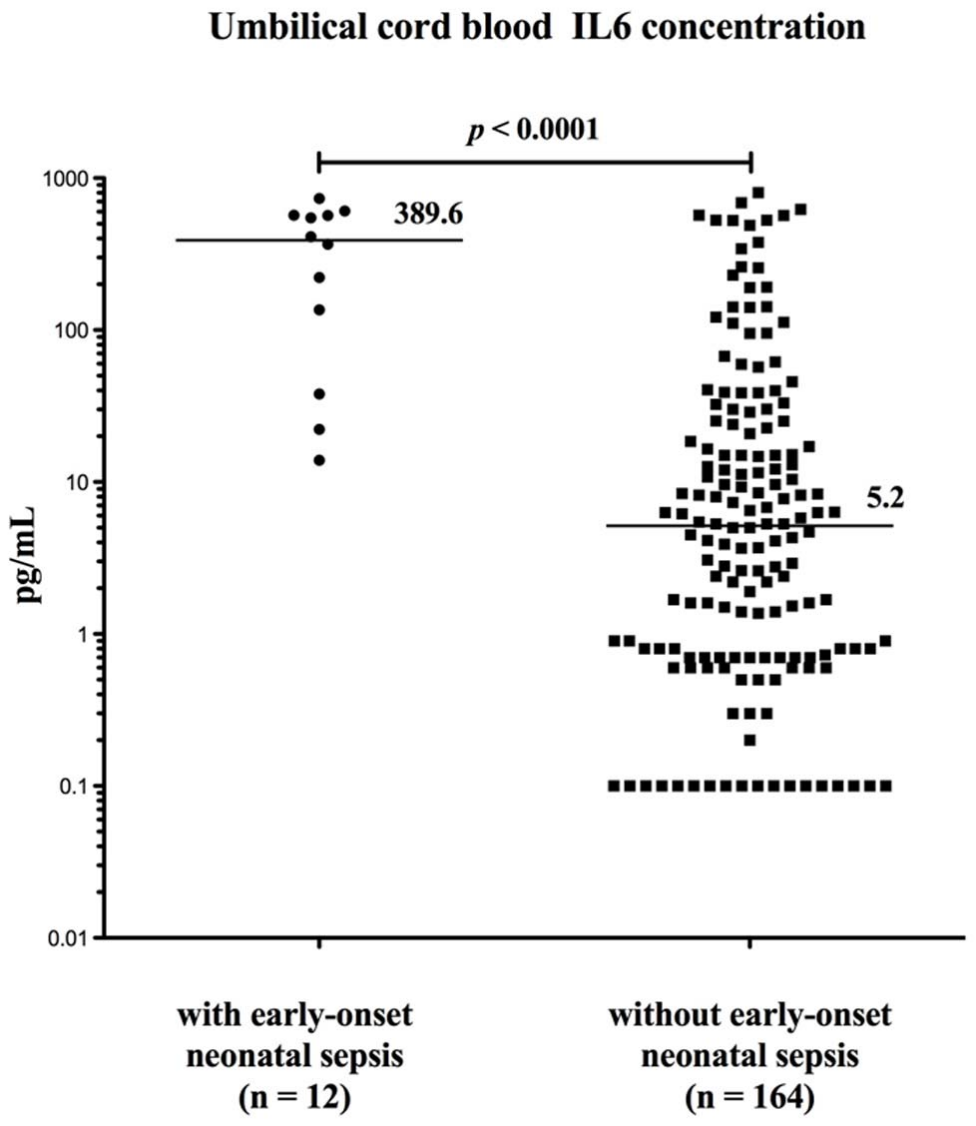
Distribution of umbilical cord blood IL6 according the occurrence of early onset neonatal sepsis.

**Figure 2:**
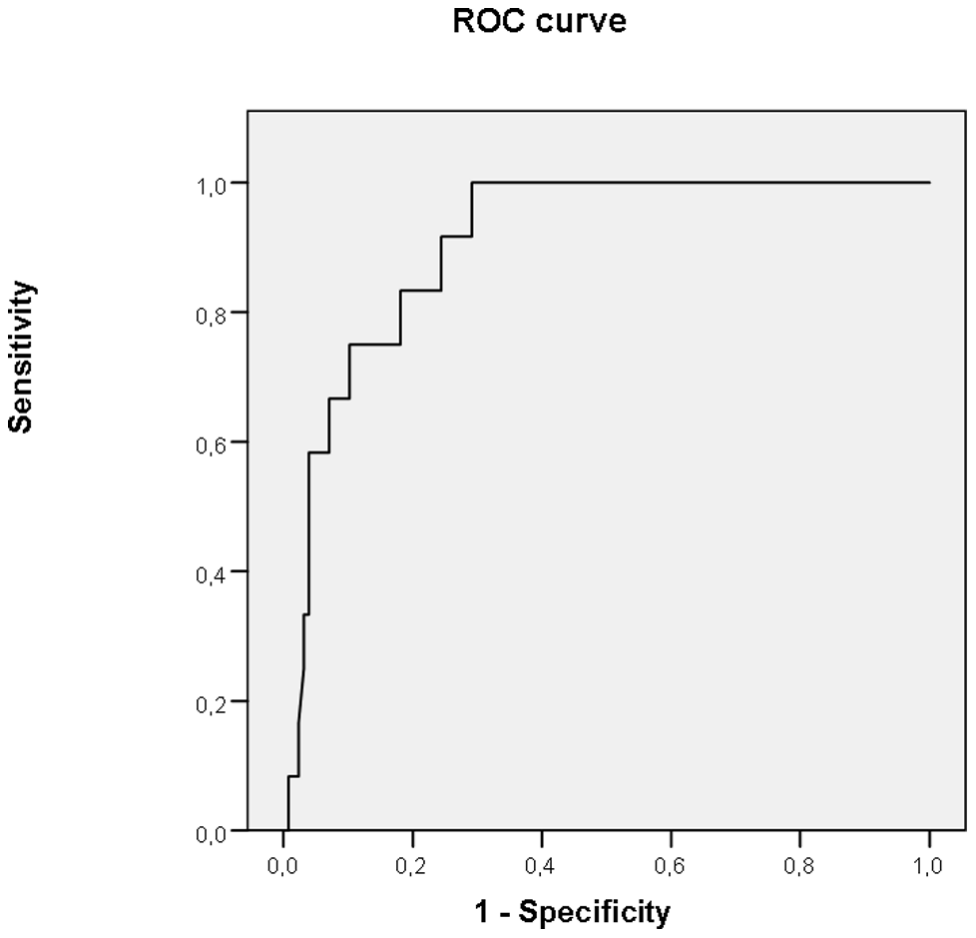
Receiver–operating characteristics (ROC) curve to display the relationship between sensitivity and false-positive rate (1 – specificity), and to select the best cut-off value for umbilical IL-6 for diagnosing early-onset neonatal sepsis.

Umbilical cord blood IL-6 levels were significantly higher in women complicated with EONS than in women without [median (range) 389.5 pg/mL (13.9–734.8) vs. 5.2 (0.1–801.4), p<0.0001; Figure 1]. Receiver operator curve analysis showed that the best cut-off value for umbilical cord IL-6 to predict EONS was 38 pg/mL (area under the curve 0.908, 95% confidence interval: 0.846–0.971) with a sensitivity of 83%, a specificity of 82%, a positive likelihood ratio of 4.6, a negative likelihood ratio of 0.2035, a positive predictive value of 30% and a negative predictive value of 98.1% (Figure 2).

Finally, logistic regression was performed to evaluate the independence of umbilical cord blood IL-6 and funisitis to predict early-onset neonatal sepsis. After adjustment by gestational age at delivery, prenatal administration of corticosteroids and antibiotics, the logistic regression indicated that the only independent predictor of early-onset neonatal sepsis was umbilical cord blood IL-6 (Table 3).

**Table 3:** Relationship between umbilical cord blood IL-6 and funisitis and early-onset neonatal sepsis analyzed by binary logistic regression.

	Odds ratio	95% Confidential interval	p
**Early-onset neonatal sepsis**			
Gestational age at delivery	0.999	0.96–1.04	0.965
Corticosteroids	1.3	0.19–8.8	0.775
Antibiotics	0.661	0.029–15.03	0.795
Umbilical cord blood IL-6 ≥38 pg/mL	13.6	2.2–81.6	0.004
Funisitis	4.1	0.86–19.8	0.074

## Discussion

Main finding of this study is that umbilical cord blood IL-6 is the only independent predictor of early-onset neonatal sepsis. Knowledge of a funisitis diagnosis in women complicated with PPROM does not add new valuable information.

The finding of fetal neutrophils into umbilical cord (funisitis) and in the chorionic plate vessels has been considered the last stage of inflammatory response associated with [20] a worse neonatal outcome [21]–[23]. In a large cohort of PPROM women classified according the presence or absence of histological chorioamnionitis with or without funisitis, our group reported a higher risk not only of EONS but also of retinopathy of prematurity in women complicated with funisitis [19]. In line with that, this study not only ratifies a higher occurrence of funisitis in women with EONS but also assesses its independence contribution as predictor of neonatal sepsis.

Regarding inflammatory biomarkers, the intensity of the fetal inflammatory response can be assessed by measuring the levels of different inflammatory mediators in umbilical cord blood. Unfortunately, there is little information regarding the dynamics of the levels of these mediators in umbilical cord blood during advanced pregnancy. The stable umbilical cord IL-6 levels during pregnancy appear to be an advantage for the description of the intensity of the fetal inflammatory response because there is no need for any adjustment for gestational age [24]–[25]. This is why umbilical cord IL-6 was selected as inflammatory biomarker in this study. Previous studies have shown that elevated fetal IL-6 levels at birth are a risk factor for EONS and other neonatal morbidities as sepsis-induced disseminated intravascular coagulation, pneumonia and cerebral palsy [26]–[31]. Our study ratifies the fact that a high fetal inflammatory response mediated by umbilical cord IL-6 was observed in newborns with EONS.

To our knowledge, there are no previous data evaluating contribution of both, umbilical cord IL-6 and funisitis, as independent biomarkers of EONS. However, there are previous references suggesting the superiority of IL-6 over other biomarkers as predictor of EONS [31], [32]. Therefore, Cernada M et al [31] compared umbilical cord IL-6 and C-reactive protein (CRP) suggesting that umbilical IL-6 was a better predictor for this neonatal outcome.

Since most neonatal morbidity is largely gestational age-dependent, this finding is clinically relevant. Regardless of gestational age at delivery and the prenatal administration of corticosteroids or antibiotics, umbilical cord IL-6 may be considered an indirect predictor of neonatal infection, at least as important as classical CRP or serum leukocytes. The retrospective histopathologic knowledge of funisitis seems not to be useful to change neonatal management.

One of the strengths of our study is that we only evaluated EONS in a specific subgroup of women (those complicated with PPROM) who had a substantially higher risk of neonatal morbidity due to infection than women without membrane ruptures or newborns born at term. Thus, there are no previous references in the literature comparing both markers of FIRS, umbilical cord blood IL-6 and funisitis, simultaneously. However, one of the limitations of the study is that it was performed in a single institution, which prevented the use of a larger sample size. Other fetal markers of infection as umbilical cord CRP have not been evaluated.

Looking to the future, the evaluation of FIRS through assessment of umbilical cord blood IL-6 level after delivery appears to be a useful predictor of EONS in preterm newborns whose mothers are complicated with PPROM even after antibiotic prophylactic treatment and fetal lung maturation.

## Conclusion

In summary, identifying umbilical cord blood IL-6 levels in women with PPROM is more important than gestational age or the presence of funisitis for predicting EONS. These results support further research to establish the potential contribution of other inflammatory biomarkers in fetal blood and non-invasive samples (such as urine) to predict neonatal morbidity.

## References

[1] Weston EJ, Pondo T, Lewis MM, Martell-Cleary P, Morin C, et al (2011) The burden of invasive early-onset neonatal sepsis in the United States, 2005-2008. Pediatr Infect Dis J Nov 30(11): 937–41. 10.1097/INF.0b013e318223bad2 PMC3193564 21654548

[2] Gomez R, Romero R, Ghezzi F, Yoon BH, Mazor M, et al (1998) The fetal inflammatory response syndrome. Am J Obstet Gynecol Jul 179(1): 194–202. 10.1016/s0002-9378(98)70272-8 9704787

[3] Romero R, Maymon E, Pacora P, Gomez R, Mazor M, et al (2000) Further observations on the fetal inflammatory response syndrome: a potential homeostatic role for the soluble receptors of tumor necrosis factor alpha. Am J Obstet Gynecol Nov 183(5): 1070–7. 10.1067/mob.2000.108885 11084543

[4] Buhimschi CS, Dulay AT, Abdel-Razeq S, Zhao G, Lee S, et al (2009) Fetal inflammatory response in women with proteomic biomarkers characteristic of intra-amniotic inflammation and preterm birth. BJOG Jan 116(2): 257–67. 10.1111/j.1471-0528.2008.01925.x PMC3791329 18947340

[5] Buhimschi CS, Buhimschi IA, Abdel-Razeq S, Rosenberg VA, Thung SF, et al (2007) Proteomic biomarkers of intra-amniotic inflammation: relationship with funisitis and early-onset sepsis in the premature neonate. Pediatr Res Mar 61(3): 318–24. 10.1203/01.pdr.0000252439.48564.37 17314690

[6] Andrys C, Drahosova M, Hornychova H, Tambor V, Musilova I, et al (2010) Umbilical cord blood concentrations of IL-6, IL-8, and MMP-8 in pregnancy complicated by preterm premature rupture of the membranes and histological chorioamnionitis. Neuro Endocrinol Lett 31(6): 857–63. 21196908

[7] Yoon BH, Romero R, Yang SH, Jun JK, Kim IO, et al (1996) Interleukin-6 concentrations in umbilical cord plasma are elevated in neonates with white matter lesions associated with periventricular leukomalacia. Am J Obstet Gynecol May 174(5): 1433–40. 10.1016/s0002-9378(96)70585-9 9065108

[8] Gotsch F, Romero R, Kusanovic JP, Mazaki-Tovi S, Pineles BL, et al (2007) The fetal inflammatory response syndrome. Clin Obstet Gynecol Sep 50(3): 652–83. 10.1097/GRF.0b013e31811ebef6 17762416

[9] Weeks JW, Reynolds L, Taylor D, Lewis J, Wan T, et al (1997) Umbilical cord blood interleukin-6 levels and neonatal morbidity. Obstet Gynecol Nov 90(5): 815–8. 10.1016/S0029-7844(97)00421-3 9351770

[10] Doellner H, Arntzen KJ, Haereid PE, Aag S, Austgulen R (1998) Interleukin-6 concentrations in neonates evaluated for sepsis. J Pediatr Feb 132(2): 295–9. 10.1016/s0022-3476(98)70448-2 9506644

[11] Wharton KN, Pinar H, Stonestreet BS, Tucker R, McLean KR, et al (2004) Severe umbilical cord inflammation-a predictor of periventricular leukomalacia in very low birth weight infants. Early Hum Dev Apr 77(1–2): 77–87. 10.1016/j.earlhumdev.2004.02.001 15113634

[12] Rovira N, Alarcon A, Iriondo M, Ibanez M, Poo P, et al (2011) Impact of histological chorioamnionitis, funisitis and clinical chorioamnionitis on neurodevelopmental outcome of preterm infants. Early Hum Dev Apr 87(4): 253–7. 10.1016/j.earlhumdev.2011.01.024 21354722

[13] Lau J, Magee F, Qiu Z, Hoube J, Von Dadelszen P, et al (2005) Chorioamnionitis with a fetal inflammatory response is associated with higher neonatal mortality, morbidity, and resource use than chorioamnionitis displaying a maternal inflammatory response only. Am J Obstet Gynecol Sep;193(3 Pt 1): 708–13. 10.1016/j.ajog.2005.01.017 16150264

[14] The Czech Society of Obstetrics and Gynecology (2007) Clinical Guidelines in Obstetrics.

[15] Salafia CM, Weigl C, Silberman L (1989) The prevalence and distribution of acute placental inflammation in uncomplicated term pregnancies. Obstet Gynecol Mar;73(3 Pt 1): 383–9. 2915862

[16] Rodwell RL, Taylor KM, Tudehope DI, Gray PH (1993) Hematologic scoring system in early diagnosis of sepsis in neutropenic newborns. Pediatr Infect Dis J May 12(5): 372–6. 10.1097/00006454-199305000-00004 8327296

[17] Rodwell RL, Leslie AL, Tudehope DI (1988) Early diagnosis of neonatal sepsis using a hematologic scoring system. J Pediatr May 112(5): 761–7. 10.1016/s0022-3476(88)80699-1 3361389

[18] Bhandari V, Wang C, Rinder C, Rinder H (2008) Hematologic profile of sepsis in neonates: neutrophil CD64 as a diagnostic marker. Pediatrics Jan 121(1): 129–34. 10.1542/peds.2007-1308 18166566

[19] Tsiartas P, Kacerovsky M, Musilova I, Hornychova H, Cobo T, et al.. (2013) The association between histological chorioamnionitis, funisitis and neonatal outcome in women with preterm prelabor rupture of membranes. J Matern Fetal Neonatal Med Apr 16. 10.3109/14767058.2013.784741 23489073

[20] Smulian JC, Bhandari V, Campbell WA, Rodis JF, Vintzileos AM (1997) Value of umbilical artery and vein levels of interleukin-6 and soluble intracellular adhesion molecule-1 as predictors of neonatal hematologic indices and suspected early sepsis. J Matern Fetal Med Sep-Oct 6(5): 254–9. 10.1002/(SICI)1520-6661(199709/10)6:5<254::AID-MFM2>3.0.CO;2-F 9360181

[21] Park CW, Moon KC, Park JS, Jun JK, Romero R, et al (2009) The involvement of human amnion in histologic chorioamnionitis is an indicator that a fetal and an intra-amniotic inflammatory response is more likely and severe: clinical implications. Placenta Jan 30(1): 56–61. 10.1016/j.placenta.2008.09.017 PMC4136806 19046766

[22] Katzman PJ, Metlay LA (2010) Fetal inflammatory response is often present at early stages of intra-amniotic infection, and its distribution along cord is variable. Pediatr Dev Pathol Jul-Aug 13(4): 265–72. 10.2350/09-02-0604-OA.1 19642812

[23] Lee SE, Romero R, Kim CJ, Shim SS, Yoon BH (2006) Funisitis in term pregnancy is associated with microbial invasion of the amniotic cavity and intra-amniotic inflammation. J Matern Fetal Neonatal Med Nov 19(11): 693–7. 10.1080/14767050600927353 17127492

[24] Matoba N, Yu Y, Mestan K, Pearson C, Ortiz K, et al (2009) Differential patterns of 27 cord blood immune biomarkers across gestational age. Pediatrics May 123(5): 1320–8. 10.1542/peds.2008-1222 19403498

[25] Mestan K, Yu Y, Thorsen P, Skogstrand K, Matoba N, et al (2009) Cord blood biomarkers of the fetal inflammatory response. J Matern Fetal Neona May 22(5): 379–87. 10.1080/14767050802609759 PMC5001950 19529994

[26] Romagnoli C, Frezza S, Cingolani A, De Luca A, Puopolo M, et al (2001) Plasma levels of interleukin-6 and interleukin-10 in preterm neonates evaluated for sepsis. Eur J Pediatr Jun 160(6): 345–50. 10.1007/pl00008445 11421413

[27] Ng PC, Li K, Leung TF, Wong RP, Li G, et al (2006) Early prediction of sepsis-induced disseminated intravascular coagulation with interleukin-10, interleukin-6, and RANTES in preterm infants. Clin Chem Jun 52(6): 1181–9. 10.1373/clinchem.2005.062075 16613997

[28] Smulian JC, Campbell WA, Vintzileos AM, Rodis JF (1997) Correlation between umbilical artery and vein levels of interleukin-6 and soluble intracellular adhesion molecule-1. J Matern Fetal Med Mar-Apr 6(2): 67–70. 10.1002/(SICI)1520-6661(199703/04)6:2<67::AID-MFM1>3.0.CO;2-N 9086419

[29] Nelson KB, Willoughby RE (2000) Infection, inflammation and the risk of cerebral palsy. Curr Opin Neurol Apr 13(2): 133–9. 10.1097/00019052-200004000-00004 10987569

[30] Lehrnbecher T, Schrod L, Rutsch P, Roos T, Martius J, et al (1996) Immunologic parameters in cord blood indicating early-onset sepsis. Biol Neonate 70(4): 206–12. 8969810 10.1159/000244366

[31] Cernada M, Badia N, Modesto V, Alonso R, Mejias A, et al (2012) Cord blood interleukin-6 as a predictor of early-onset neonatal sepsis. Acta Paediatr May 101(5): e203–7. 10.1111/j.1651-2227.2011.02577.x 22211677

[32] Fan Y, Yu JL (2012) Umbilical blood biomarkers for predicting early-onset neonatal sepsis. World J Pediatr May 8(2): 101–8. 10.1007/s12519-012-0347-3 22573419

